# Dysfunctional decidual CD2^+^CD4^+^T cells regulated by Rev-erbα - GFPT1 - GPI anchored CD58 axis of decidual stromal cells underlies sleep disturbance induced recurrent pregnancy loss

**DOI:** 10.3389/fimmu.2026.1703925

**Published:** 2026-02-18

**Authors:** Mingke Qiu, Junyi Zhang, Yujie Luo, Xinhang Meng, Songcun Wang, Liyuan Cui

**Affiliations:** 1 Department of Interventional Vascular Surgery, Xinhua Hospital, Shanghai JiaoTong University, School of Medicine, Shanghai, China; 2 Laboratory for Reproductive Immunology, Obstetrics & Gynecology Hospital of Fudan University, Shanghai, China; 3 Obstetrics & Gynecology Hospital of Fudan University, Shanghai Key Lab of Reproduction and Development, Shanghai Key Lab of Female Reproductive Endocrine Related Diseases, Shanghai, China; 4 Shanghai Jiai Genetic & IVF Institute, Obstetrics & Gynecology Hospital of Fudan University, Shanghai, China

**Keywords:** CD2, GFPT1, glycosylphosphatidylinositol, recurrent pregnancy loss, sleep disturbance

## Abstract

**Background:**

The multifactorial nature of recurrent pregnancy loss (RPL) requires a comprehensive understanding of its diverse risk factors, including sleep disturbance. Previously, we reported decreased Rev-erbα expression in decidual stromal cells from pregnant mice with sleep disturbance (SD) and patients of RPL with sleep disturbance (RS).

**Methods:**

Omics analyses were used to predict the interaction between Rev-erbα and glutamine-fructose-6-phosphate amidotransferase 1 (GFPT1). Tunicamycin and peptide-N-glycosidase F and phosphatidylinositol-specific phospholipase C treatment were conducted to analyze the glycosylation modification type of CD58. The coculture between decidual immune cells and decidual stroma cells (DSCs) in which Rev-erbα, GFPT1, or CD58 was either knockdown or overexpressed was performed to evaluate the crosstalk of CD58^+^DSCs and CD2^+^CD4^+^T cells. Mouse model with sleep disturbance was established to explore the effect of Rev-erbα - GFPT1 - CD58 - CD2 axis on pregnancy.

**Results:**

We found that glycometabolism related GFPT1, which regulated by Rev-erbα, increased CD58 expression via glycosylphosphatidylinositol modification not classical N- or O-linked glycosylation modification, leading to the disorder of decidual CD2^+^CD4^+^T cells and consequently, resulting in miscarriage. Anti-CD2 antibody significantly decreased the risk of abortion of SD mice, proving a potential therapeutic target for adverse pregnancy outcomes induced by circadian rhythm disruption.

**Conclusions:**

Rev-erbα - GFPT1 - CD58 - CD2 axis played important role in pregnancy maintenance by regulating the crosstalk between DSCs and decidual CD4^+^T cells. Given that poor sleep is a common problem during pregnancy, our findings may have important implications for understanding the etiology of RPL and developing targeted therapy of RPL. In addition, we should pay attention to the sleep health of pregnant women, especially those with a history of RPL.

## 1 Introduction

The occurrence of recurrent pregnancy loss (RPL) is still a major challenge for reproductive medicine as RPL not only brings significant physical and psychological stress to both husband and wife, but it also imposes a heavy burden on family, social, and public health systems. RPL is a distressing condition defined by the two or more consecutive miscarriages before the fetus reaches viability, and 5% of women experience this pain when they are pregnant (1, 2). In addition, women with a history of RPL seem to have higher risks for pregnancy loss, placental problems, hypertensive pregnancy disorders, and preterm birth in the subsequent pregnancy (3, 4). Exploring the underlying mechanisms of RPL and identifying its therapeutic targets are imperative.

Routinely, the management of RPL patients is restricted to the investigation and treatment of a limited number of the known causes, such as chromosomal abnormalities, anatomical pathologies, endocrine dysfunctions, and autoimmunological factors. Based on the recommendations from the main international guidelines, the pathogenesis of RPL is determined in only almost 50% of women of the patients. Accordingly, approximately 50% of RPL causes are still unexplained, and the condition is termed as idiopathic or unexplained (2, 5). The multifactorial nature of RPL requires a comprehensive understanding of its diverse risk factors.

Pregnancy involves decidualization, blastocyst implantation, placentation and finally birth of offspring. The success of each event is essential to advance toward the next stage (6). During normal pregnancy, human extravillous trophoblast cells invade into the maternal decidua, dissolve the extracellular matrix, remodel the uterine vasculature and come into direct contact with the maternal decidual immune cells (7). The maternal immune system provides competent responses to infections as well as accepts the fetus expressing allogeneic paternal antigens (8). Aberrant implantation, defective decidualization and imbalance of maternal-fetal tolerance have been found to be closely related to RPL (8–11). Any risk factors contributed to these processes should be taken seriously.

Poor sleep is widely recognized as a common complaint during pregnancy. Sleep disturbance might cause miscarriage by changing circadian gene expression (12, 13). Previously, we reported decreased Rev-erbα (also known as nuclear receptor subfamily 1 group D member 1, NR1D1) expression in decidual stromal cells (DSCs) from pregnant mice with sleep disturbance (SD) and patients of RPL with sleep disturbance (RS). Circadian gene Rev-erbα influenced by sleep conduced to pregnancy by promoting endometrial decidualization (14) and by suppressing lipopolysaccharide (LPS)-induced macrophage M1 polarization (15). Recent studies indicated circadian disruption induced glycosylation modification disorder (16). Glycosylation modification of proteins played important roles in decidualization (17, 18). However, whether glycosylation modification affected by sleep regulated maternal-fetal tolerance to maintain pregnancy remained unclear.

In the present study, we screened the differentially expressed genes (DEGs) between primary human endometrial stromal cells (ESCs) with and without induced decidualization *in vitro*. The DEGs enriched in circadian rhythm related and glycometabolism related signaling pathways by Gene ontology (GO) analysis. Glycobiological and glycosylation studies have also shed light on the molecular mechanisms involved in pregnancy loss (19, 20). Could Rev-erbα affect pregnancy outcomes by regulating the glycometabolism? If so, what is the mechanism? With these questions in mind, we explored the common core genes in Rev-erbα regulated genes and in glycometabolism. In addition, with omics analyses, functional experiments and SD mouse model studies, we conducted that Rev-erbα - glutamine-fructose-6-phosphate amidotransferase 1 (GFPT1) - CD58 - CD2 axis played important role in pregnancy maintenance by regulating the crosstalk between DSCs and decidual CD4^+^T (dCD4^+^T) cells.

## 2 Materials and methods

### 2.1 Human samples

Human endometrial tissues during secretory phase were collected from women with regular menstrual cycles who did not have underlying endometrial abnormalities and did not receive exogenous steroidal hormones therapy for three months preceding biopsy collection. Human decidual tissues (gestational age: 6–12 weeks) were obtained from healthy pregnancies who were aged between 22 and 40 and artificially terminated for non-medical reasons or miscarriages who were diagnosed as recurrent pregnancy loss (RPL), and excluding cases attributed to endocrine, anatomic, genetic abnormalities, infection, etc. All participants were required to complete the questionnaire of patients pittsburgh sleep quality index (PSQI). Participants with PSQI ≤ 5 were considered to have normal sleep, Participants with PSQI > 5 were considered to have sleep disturbance (**Table 1**). Written informed consent was obtained from all participants.

**Table 1:** Clinical characteristics of enrolled subjects.

Subjects	NN	RS	P value
Number	52	24	–
Age mean(years) ^a^	29.65 ± 0.59	30.21 ± 0.90	ns
Gestational age(week)	7.27 ± 0.12	7.18 ± 0.18	ns
Previous pregnancy losses(number)	–	2.54 ± 0.13	ns
PSQI score	0.52 ± 0.15	8.41 ± 0.36	<0.0001

NN, Normal pregnancy with normal sleep; RS, Patients of recurrent pregnancy loss with sleep disturbance; PSQI, Pittsburgh sleep quality index; NS, No significance (ns). Pregnant termination in NN group is for non-medical reasons. Recurrent pregnancy loss in RS group refers to unexplained pregnancy loss, excluding those resulting from endocrine, anatomic, genetic abnormalities, infection, etc. ^a^ Mean *±* standard error of the mean (SEM).

Human endometrial tissues were digested with 1.0 mg/mL collagenase IV (C5138, Sigma-Aldrich) to obtain endometrial stromal cells (ESCs) and they were cultured in complete medium (Dulbecco’s modified Eagle’s medium/F-12 (DMEM/F12 supplemented with 10% fetal bovine serum, 100 U/mL penicillin and 100 μg/mL streptomycin) as described previously (14). Decidual stromal cells (DSCs) and decidual immune cells (DICs) were separated from decidual tissues after digestion with 1.0 mg/mL collagenase IV (C5138, Sigma-Aldrich) and 150 U/mL DNase I in DMEM/F12 and density gradient centrifugation with percoll as described previously (21).

### 2.2 Cell treatment

For *in vitro* decidualization, ESCs were treated with 1 μM MPA and 0.5mM cAMP (T1418, Topscience, Shanghai, China) in complete medium for 48 h. For si-RNA or plasmid transfection, DSCs were dealt with Rev-erbα/glutamine-fructose-6-phosphate amidotransferase 1(GFPT1)/O-GlcNAc transferase (OGT) specific siRNA (Ribobio, China) or Rev-erbα/GFPT1 plasmid (Public Protein/Plasmid Library, China) for 20 h using transfection reagent (L3000015, Invitrogen or 301425, QIAGEN, Germany) according to the manufacturer’s instructions. In some experiments, DSCs were treated with tunicamycin (1 μM) for 48h. Cell lysates were incubated with tunicamycin and peptide-N-glycosidase F (PNGaseF) (Roche, Germany) at 37 °C for 1 h. DSCs were incubated with phosphatidylinositol-specific phospholipase C (PI-PLC) (P6466, Thermo Fischer Scientific) or 0.25% glycerol (negative control) in PI-PLC buffer at 37 °C for 1.5 h.

For the co-culture of decidual immune cells and DSCs, Freshly isolated DSCs with or without indicated treatments were seeded at a density of 2×10^5^ cells/ml per well in 24-well plates overnight. The cells were then washed with PBS (HyClone, U.S.A.). Equal numbers of decidual immune cells were added to each well. PMA (50 ng/ml), ionomycin (1 μg/ml) and brefeldin A (10 mg/ml) were added 4h before the end of the 48h culture for intracellular cytokine analysis. The immune cells were then harvested for flow cytometry analysis.

For cycloheximide (CHX) chase assay, DSCs were transfected with *si-GFPT1* for 48 h. The cells were treated with CHX (50 μg/mL) for indicated times (1, 2, 4 hours) before cell lysate collection. The CD58 protein levels were quantified by immunoblot.

### 2.3 RNA-Seq data analysis

The total RNA of ESCs with or without *in vitro* decidualization was extracted using TRIzol reagent (Qiagen, Germany). mRNA was enriched from total RNA followed by a cDNA library construction, and then sequenced on the BGISEQ-500 sequencing platform (BGI-shenzhen Technology Co., Ltd). There are four biological replicates in RNA-seq. |Log_2_ fold change| > 0.2630 along with P-value < 0.05 were used as criteria for defining differential expression in RNA-seq.

### 2.4 ChIP-seq

The ChIP assay was performed following the manufacturer’s instructions (Millipore, Billerica, USA). ESCs were placed in 1% formaldehyde for 10 min to cross-link the DNA, and the cross-linking was quenched using 2.5M glycine solution. The cells were then homogenized with PBS containing protease inhibitors. The supernatant was collected after centrifugation and added to CHIP dilution buffer, and the mixture was divided into two portions: the positive control input group, the experimental group with Rev-erbα antibody (13418, CST). The enriched DNA fragments from three biological replicates were purified and used to construct libraries. Subsequently, the libraries were subjected to sequencing using the Illumina novaseq6000 (Sangon biotech, China). The raw data obtained from ChIP-seq was filtered to obtain clean reads and further analyzed using Bowtie2, MEME and MAnorm2 software. During the filtering process, peaks with an M value greater than 1 or less than -1 and a P value less than 0.01 were considered as differentially modified peaks.

### 2.5 Mass spectrometric analysis

Protein lysates with or without *GFPT1* knockdown were treated with 25mM dithiothreitol (DTT) to achieve a final concentration of approximately 10mM. The protein concentrations were detected using Bradford method. After proteolysis and DDA library construction, the peptides were subsequently subjected to the LC-MS analysis by BGl Tech Solutions Co.,Ltd (Shenzhen, China). Proteins were discovered using the Uniprot Homo sapiens database. There are three biological replicates. |Log2 fold change| > 0.2630 along with P-value < 0.05 were used as criteria for defining differential expression.

### 2.6 Western blot

The tissue and cell samples were lysed with cold radio-immunoprecipitation (RIPA) buffer (Beyotime Biotechnology, China) supplemented with a protein inhibitor cocktail (MCE, China) and a phosphatase inhibitor cocktail (MCE, China). Protein concentrations were determined by the BCA method. Lysates were heated at 100 °C for 10 min, and then loaded on 10% gels (Bio-Rad, U.S.A) for SDS-polyacrylamide gel electrophoresis. After electrophoretic separation, the proteins were transferred onto 0.2 μm PVDF membranes (Amersham, Germany), blocked with 5% nonfat milk, and incubated overnight at 4 °C with the primary antibodies targeting: anti- Rev-erbα (sc-393215, Santa Cruze), anti-GFPT1 (ab125069, Santa Cruz Biotechnology, U.S.A), anti-CD58 (ab275392, Abcam, U.S.A), anti-CD48 (83871-1-RR, proteintech, USA), anti-Tubulin (ab179513, Abcam, U.S.A) and anti-ACTB (ab16769, Abcam, U.S.A). β-Tubulin and ACTB were used as internal standards. Membranes were washed and incubated with HRP conjugated secondary antibody (Jackson, U.S.A) at room temperature for 1h. The antibody-labeled proteins were detected by chemiluminescence using Chemiluminescent HRP Substrate (Millipore, U.S.A), in an Amersham™ Imager 600 (GE Healthcare, U.S.A).

### 2.7 Mice

All C57 BL/6 mice (6–8 weeks) were purchased from GemPharmatech Co., Ltd. Mice were bred in a room of 22-25°C, 40-60% relative humidity, 12 h light-12 h dark cycles with the same time of light-on every day and fed with food and water ad libitum. For sleep disturbance model, the mice were raised in room of 12 h light-12 h dark cycles with different time of light-on. The time of light-on (referred to ZT0) was advanced 6 h every four days for 3 months. The female mice and male mice were caged together at 19:00, and the vaginal plugs were detected at next 7:00, which referred to embryonic 0.5 days (E0.5). Mice with sleep disturbance were divided into two groups. One group of mice were injected intraperitoneally with 10 μg CD2 antibody (100119, Biolegend) at E3.5 and E8.5. Another group of mice were injected intraperitoneally with equivalent physiological saline at E3.5 and E8.5, alongside a control group that had normal sleep. All mice were sacrificed by trained staff and accomplished using 5% isoflurane followed by cervical dislocation at E13.5 to observe the pregnancy outcomes. The percentage of fetal loss (the embryo absorption rate) was calculated using the following formula: % of resorption = R/(R+V) ×100, where R represents the number of hemorrhagic implantation sites (sites of fetal loss) and V stands for the number of viable, surviving fetuses.

Uteri from pregnant mice were dissected to remove the mesometrium and were excised at the ovaries and cervix. The fetal and placental tissues were carefully removed and rinsed in PBS. Minced uteri were digested in RPMI 1640, supplemented with collagenase type IV and DNase I for 45 min at 37 °C with gentle agitation. The resulting cells were cultured in RPMI 1640 enriched with 10% FBS, 100U/mL penicillin, 100 μg/mL streptomycin, and 1 μg/mL amphotericin B at 37 °C in an atmosphere of 5% CO_2_ for 4 h, allowing for the selective detachment of adherent stromal cells. PMA (50 ng/mL, Biolegend, U.S.A.), ionomycin (1 μg/mL, Biolegend, U.S.A.) brefeldin A (10 mg/mL, BioLegend, U.S.A.), were added to the cell cultures and incubated for 4 h for intracellular cytokine analysis of CD4^+^T cells.

### 2.8 Flow cytometry

Cell surface molecular expression and intracellular cytokine production were evaluated using flow cytometry. A panel of fluorochrome-conjugated antibodies was employed to detect specific markers: Brilliant Violet 605-conjugated anti-human CD4, FITC-conjugated anti-human IL-17A, anti-mouse IL-4; PE/CY7-conjugated anti-human TGF-β1, anti-mouse TNFα, IL-17A; PerCP/Cy5.5-conjugated anti-human IL-4; Brilliant Violet 421-conjugated anti-mouse TGF-β1; Brilliant Violet 510-conjugated anti-human TNFα, IFNγ, anti-mouse CD4; ECD-conjugated anti-mouse IFNγ (Biolegend, U.S.A.) antibodies. For intracellular staining, cells were fixed and permeabilized using the Fix/Perm kit (Biolegend, U.S.A.). Flow cytometry was performed on a Beckman-Coulter CyAn ADP cytometer (Beckman-Coulter, U.S.A.) and analyzed with FlowJo software (Tree Star, Ashland, U.S.A.).

### 2.9 Statistical analysis

Data are tested for normal distribution (Kolmogorov-Smirnov), defining whether the results should be analyzed parametrically or non-parametrically. For the normally distributed data, significance of differences between two groups was determined by Student’s t-test. For the non-normally distributed data, significance of differences between two groups was determined by Mann-Whitney-test. Multiple groups were analyzed by one-way ANOVA with the *post-hoc* Dunnett t-test using Prism Version 8 software (GraphPad, San Diego, CA, USA). Variables were presented as means and standard error of mean (SEM). For all statistical tests, p- values <0.05 were considered statistically significant.

## 3 Results

### 3.1 Rev-erbα regulated glycometabolism-related GFPT1 expression of decidual stromal cells

**Figure 1:**
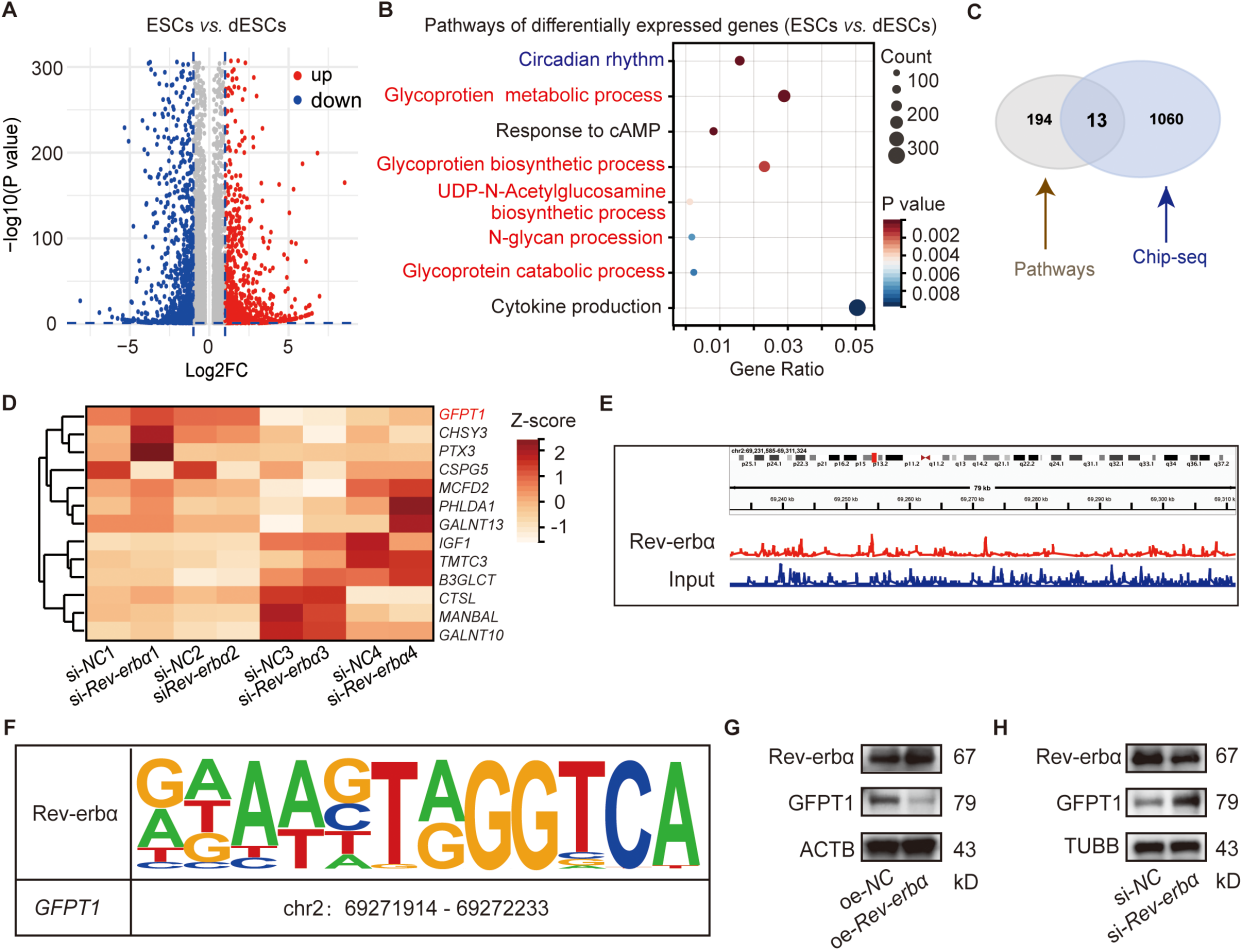
Rev-erbα regulated glycometabolism related GFPT1 expression of decidual stroma cells (DSCs). **(A)** The volcano plot of the differential mRNAs between human primary endometrial stromal cells (ESCs) and *in vitro* decidualized ESCs (dESCs) (n=4). **(B)** Enriched Gene Ontology term pathways of differential gene clustering between ESCs and dESC. **(C)** There are 13 glycometabolism related genes [from **(B)**] could also bound to Rev-erbα (from the chromatin immunoprecipitation sequencing (ChIP-seq) analysis). **(D)** The differential expression of the 13 genes [from **(C)**] of *Rev-erbα* knockdown dESCs and control dESCs. **(E)** The binding of *GFPT1* genes to *Rev-erbα* in ChIP-Atlas. **(F)** Prediction of the binding sites between *Rev-erbα* and *GFPT1* via JASPAR. **(G, H)** The protein level of Rev-erbα and GFPT1 in DSCs after indicated treatments examined by western blot. Images are representatives of three independent experiments.

We conducted microarray profiling on human primary endometrial stromal cells (ESCs) and *in vitro* decidualized ESCs (dESCs). Unique gene signatures for ESCs and dESCs were generated based on 1.2-fold differential expression between the two different ESCs population. These commonly differentially expressed probes from ESCs and dESCs were highlighted on the combined data sets and presented as a volcano plot (**Figure 1A**). The enrichment analysis of the Gene Ontology (GO) term showed that the differentially expressed genes (DEGs) were enriched in circadian rhythm and glycometabolism-related (glycoprotein metabolic process, UDP-N-Acetylglucosamine biosynthetic process, N-glycan procession, and so on) signaling pathways (**Figure 1B**). Previously, we reported that circadian gene Rev-erbα could regulate decidualization and maternal-fetal tolerance (14, 15). To verify whether Rev-erbα, as a transcription factor, could affect pregnancy outcomes by regulating the glycometabolism, we employed a combination of the RNA-seq and chromatin immunoprecipitation sequencing (ChIP-seq) analysis to identify the DEGs of Rev-erbα regulated glycometabolism-related genes. As shown in **Figure 1C**, there are 13 glycometabolism-related genes from **Figure 1B** could also bound to Rev-erbα. Rev-erbα is an important circadian gene and also functions as transcriptional repressor (22). Among the 13 genes, only *GFPT1, PHLDA1* and *PTX3* expression of DSCs were upregulated after with si-*Rev-erbα* transfection (**Figure 1D**). Glutamine-fructose-6-phosphate amidotransferase 1 (GFPT1) attracted our interests as GFPT1 is the rate-limiting enzyme of hexosamine biosynthesis pathway and plays a role in the development of tumor (23). ChIP-seq results demonstrated the combination of *GFPT1* and *Rev-erbα* (**Figures 1E, F**). In addition, *Rev-erbα* overexpression significantly decreased GFPT1 protein expression of decidual stromal cells (DSCs) (**Figure 1G**), while *Rev-erbα* knockdown increased GFPT1 protein expression of DSCs (**Figure 1H**). Thus, Rev-erbα could regulate glycometabolism-related GFPT1 expression of DSCs.

### 3.2 GFPT1 increased CD58 expression via glycosylphosphatidylinositol modification

**Figure 2:**
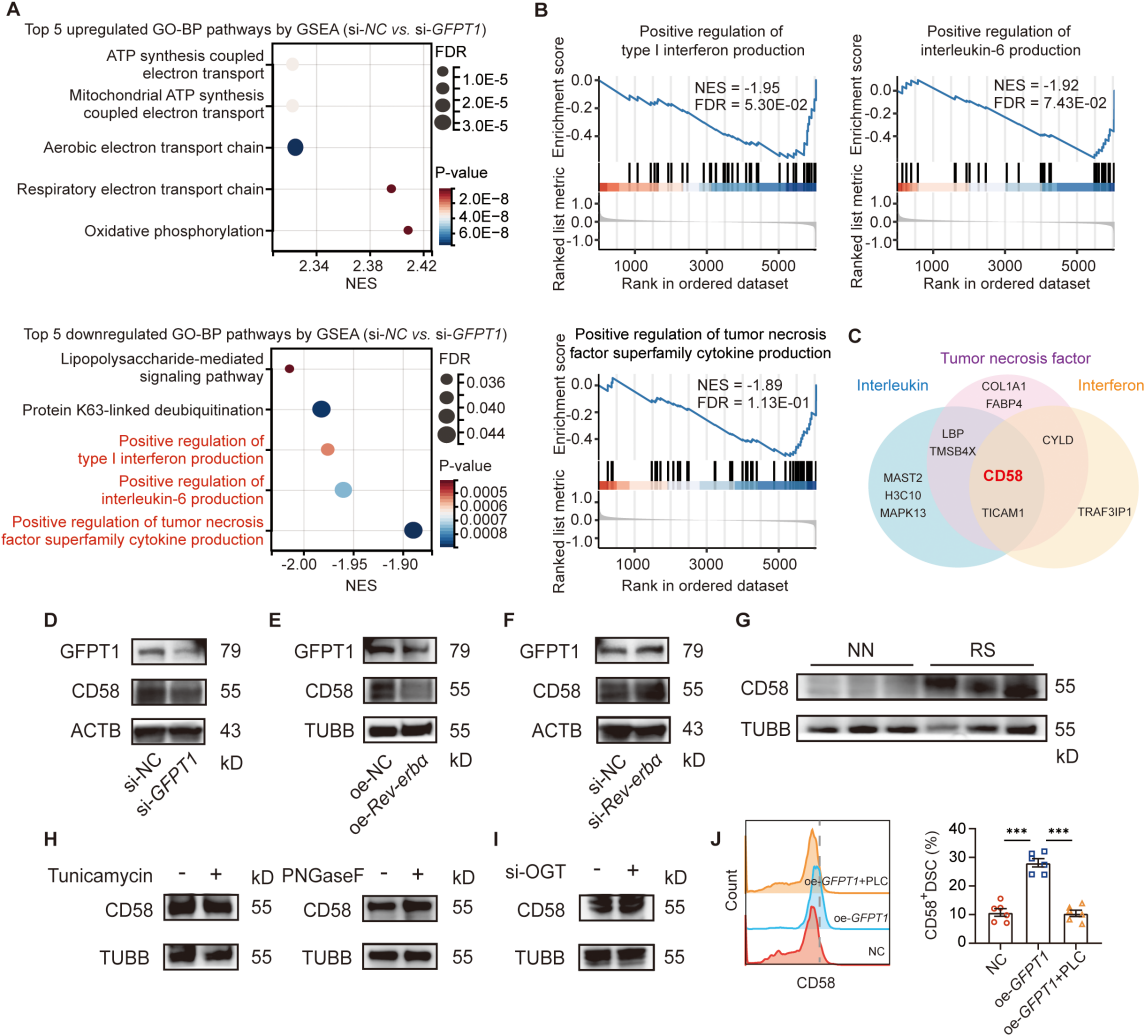
GFPT1 increased CD58 expression via glycosylphosphatidylinositol (GPI) modification. **(A)** The top 5 upregulated and top 5 downregulated GO pathways by GSEA between DSCs with and without *GFPT1* knockdown. **(B)** GSEA showing differentially enriched immune pathways between DSCs with and without *GFPT1* knockdown. **(C)** Two key proteins (CD58 and TICAM1) were mapped simultaneously into the regulation of interferon, interleukin, and tumor necrosis factor superfamily cytokine production pathways. **(D–F)** The protein level of GFPT1 and CD58 in DSCs after indicated treatments examined by western blot. **(G)** The protein level of CD58 in the DSCs from normal pregnancy with normal sleep (NN) and recurrent pregnancy loss (RPL) with sleep disturbance (RS) examined by western blot. **(H, I)** The protein level of CD58 in DSCs after indicated treatments examined by western blot. **(J)** Quantification of CD58 staining in DSCs after indicated treatments. Images are representative of three individual experiments (n=6, Treatment groups were compared via Student’s t-test). Data represent the mean ± SEM. The data points represent the biological replicates obtained from three independent experiments. ***p<0.001.

To investigate the role and mechanism of GFPT1 in pregnancy maintenance, we screened the differentially expressed proteins between DSCs with and without *GFPT1* knockdown. As shown in **Figure 2A** and **2B**, the differentially expressed proteins were enriched in immune regulation related pathways (such as positive regulation of type I interferon production, interleukin-6 production, and tumor necrosis factor superfamily cytokine production, and so on) by GO and gene set-enrichment analysis datasets (GSEA). Two key proteins (CD58 and TICAM1) were mapped simultaneously into the regulation of interferon, interleukin, and tumor necrosis factor superfamily cytokine production pathways (**Figure 2C**). Given that CD58 is a heavily glycosylated, distributed surface glycoprotein (24), and GFPT1 plays important role in protein glycosylation (25, 26), we choose CD58 as the research candidate in the follow-up experiments. *GFPT1* knockdown decreased CD58 protein expression of DSCs (**Figure 2D**). Cycloheximide (CHX) chase assay showed the increased degradation of CD58 in DSCs with GFPT1 knockdown (**Supplementary Figure 1**). *Rev-erbα* overexpression significantly decreased GFPT1 and CD58 expression (**Figure 2E**), while *Rev-erbα* knockdown increased GFPT1 and CD58 expression of DSCs (**Figure 2F**). In addition, the increased expression of CD58 were also observed in DSCs from patients of recurrent pregnancy loss (RPL) with sleep disturbance (RS) compared to those from normal pregnancy with normal sleep (NN), suggesting that CD58, which was regulated by GFPT1 and Rev-erbα, was associated with miscarriage of sleep disturbance (**Figure 2G**).

However, peptide-N-glycosidase F (PNGaseF) treatment, which inhibits N-linked glycosylation and removes the N-glycan from the protein respectively, had no effect on CD58 expression of DSCs (**Figure 2H**). O-GlcNAc transferase (OGT) knockdown did not interfere CD58 expression of DSCs (**Figure 2I**). These results inspired us to think whether GFPT1 regulated CD58 expression of DSCs by other modification. CD58 also has a glycosylphosphatidylinositol (GPI) anchored form (24), and GFPT signal was reported to play a role in the depletion of GPI precursors (27). Interestingly, phosphatidylinositol-specific phospholipase C (PI-PLC), which cleaves GPI anchored proteins, reversed the increase CD58 expression induced by *GFPT1* overexpression (**Figure 2J**). These data indicated that GFPT1 might increase CD58 expression by GPI-modification.

### 3.3 Rev-erbα-GFPT1-CD58-CD2 axis played an important role in maternal-fetal tolerance by regulating cytokine profile of dCD4^+^T cells

**Figure 3:**
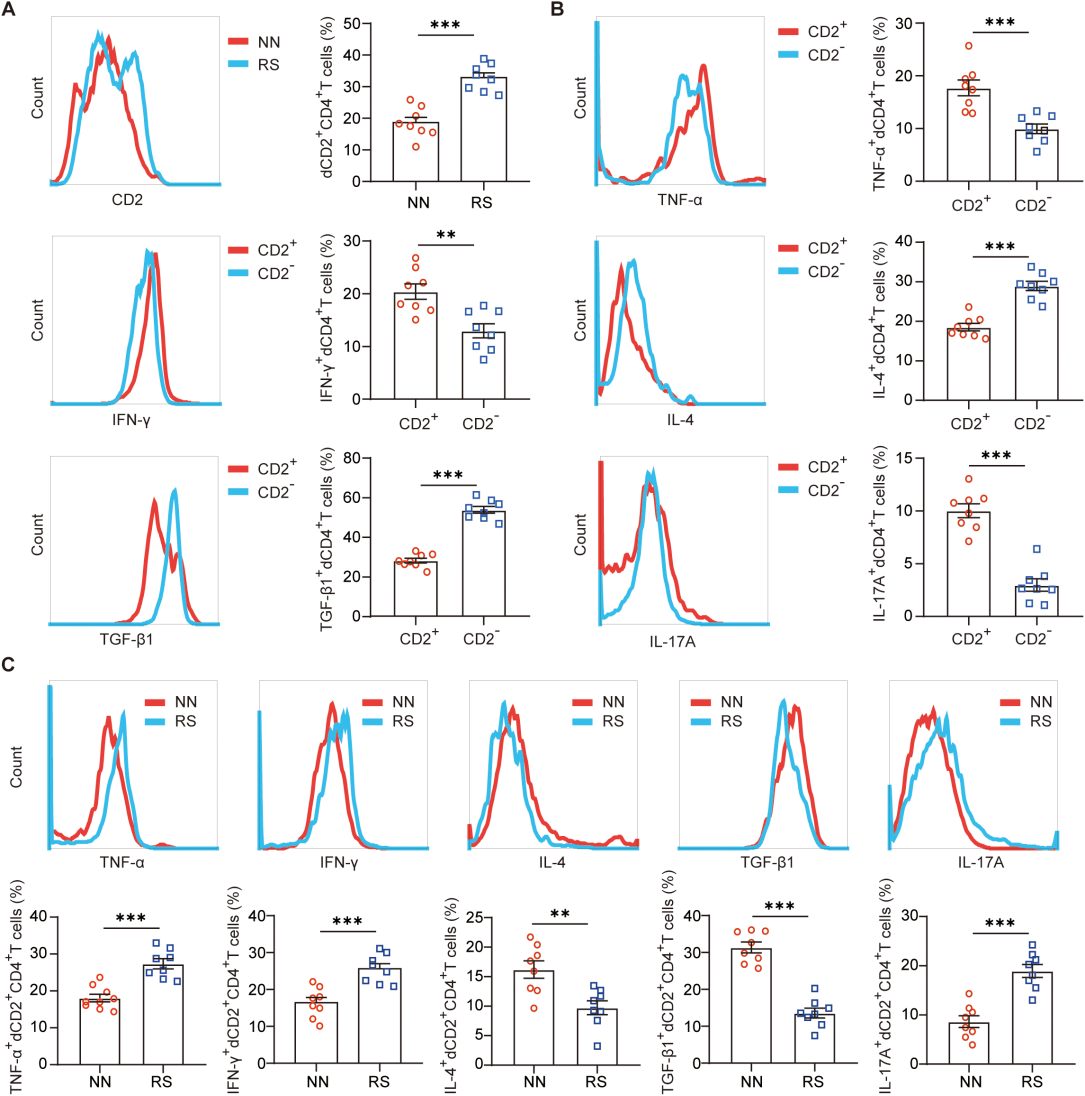
Altered frequency and function of dCD2^+^CD4^+^T cells in RS patients. **(A)** Frequency of CD2 expressing cells in gated CD4^+^ T cells from decidual immune cells from NN and RS as determined by flow cytometric analysis (n=8, Treatment groups were compared via Student’s t-test). **(B)** Quantitation of flow cytometric analysis of Th1(TNF-α and IFN-γ), Th2 (IL-4), Treg (TGF-β1) and Th17 (IL-17A)- type cytokine expression of dCD2^+^CD4^+^T cells (n=8, Treatment groups were compared via Student’s t-test). **(C)** Cytokine expression on dCD2^+^CD4^+^T cells from NN and RS was assessed by flow cytometric analysis (n=8, Treatment groups were compared via Student’s t-test). A representative dot plot is also shown. Data represent the mean ± SEM. The data points represent the biological replicates obtained from three independent experiments. **P<0.01, ***P<0.001. NN, normal pregnancy with normal sleep; RS, patient of RPL with sleep disturbance.

CD58 is also known as lymphocyte-function antigen 3, CD2 is the natural ligand of CD58, and CD2-CD58 interaction is a crucial costimulatory signal in the modulation of T cell responses (28). For many years, it has been believed that a shift in the maternal immune response towards a Th2 bias and Treg expansion is crucial for maintaining a successful pregnancy (8). We wondered whether higher CD58 expression of DSCs influenced maternal-fetal tolerance of RS via CD2-CD58 interaction? Higher CD2 expression of decidual CD4^+^T (dCD4^+^T) cells were also observed in RS patients compared to those from NN (**Figure 3A**). Next, we evaluated whether CD2 expression correlated with the dCD4^+^ T cells function of producing cytokines. We found that the expression of pro-inflammatory cytokines (TNF-α, IFN-γ, and IL-17A) by dCD2^+^CD4^+^T cells was more than that by dCD2^-^CD4^+^T cells, while dCD2^+^CD4^+^T cells are associated with decreased Th2- and Treg-type cytokines expression (**Figure 3B**). These data gave the first indication that dCD4^+^T cells characterized by differential expression of CD2 contained cells in different functional states. Additionally, dCD2^+^CD4^+^T cells from RS expressed higher levels of TNF-α, IFN-γ and IL-17A, but lower amounts of IL-4, TGF-β1 than that from NN (**Figure 3C**).

**Figure 4:**
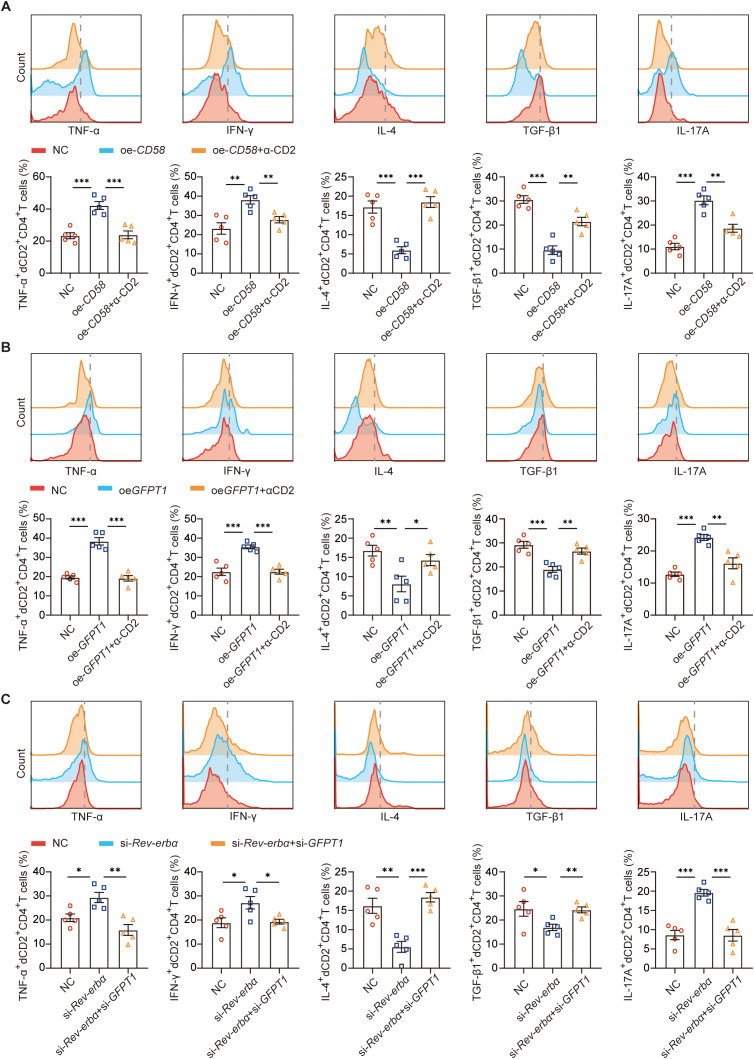
Rev-erbα-GFPT1-CD58-CD2 axis played an important role in maternal-fetal tolerance by regulating cytokine profile of dCD4^+^T cells. **(A)** Quantitation of flow cytometric analysis of TNF-α, IFN-γ, IL-4, TGF-β1 and IL-17A expression on dCD2^+^CD4^+^T cells in the coculture system of decidual immune cells and DSC with or without *CD58* overexpression, in the presence or absence of anti-CD2 antibody (n=5, Treatment groups were compared via Student’s t-test). **(B)** Cytokine expression on dCD2^+^CD4^+^T cells in the coculture system of decidual immune cells and DSC with or without *GFPT1* overexpression, in the presence or absence of anti-CD2 antibody (n=5, Treatment groups were compared via Student’s t-test). **(C)** Cytokine expression on dCD2^+^CD4^+^T cells in the coculture system of decidual immune cells and DSCs with or without *Rev-erbα* knockdown, or DSCs with *Rev-erbα* and *GFPT1* knockdown. Images are representative of three individual experiments. Data represent the mean ± SEM (n=5, Treatment groups were compared via Student’s t-test). The data points represent the biological replicates obtained from three independent experiments. *p<0.05, **p<0.01, ***p<0.001.

Then, we established co-culture systems of DSCs and dCD4^+^T cells to determine whether altered Rev-erbα-GFPT1-CD58 axis of DSCs could contribute to the disorder of dCD2^+^CD4^+^T cells. As shown in **Figure 4A** and **4B**, both *CD58* or *GFPT1* overexpression of DSCs could increase TNF-α, IFN-γ and IL-17A, but decrease IL-4, TGF-β1 expression of dCD2^+^CD4^+^T cells, while CD2 blocking antibody reversed their effects. Furthermore, *Rev-erbα* knockdown, which increased GFPT1 and CD58 expression of DSCs, also promoted the pro-inflammatory cytokines, but inhibited the anti-inflammatory cytokines expression of dCD4^+^T cells. And as expected, simultaneously *GFPT1* knockdown of DSCs essentially eliminated the effects of *Rev-erbα* knockdown (**Figure 4C**). These data suggested that Rev-erbα-GFPT1-CD58-CD2 axis might play an important role in maternal-fetal tolerance by regulating cytokine profile of decidual CD4^+^T cells.

### 3.4 Effects of blockade of CD2 on pregnancy of mice with sleep disturbance

**Figure 5:**
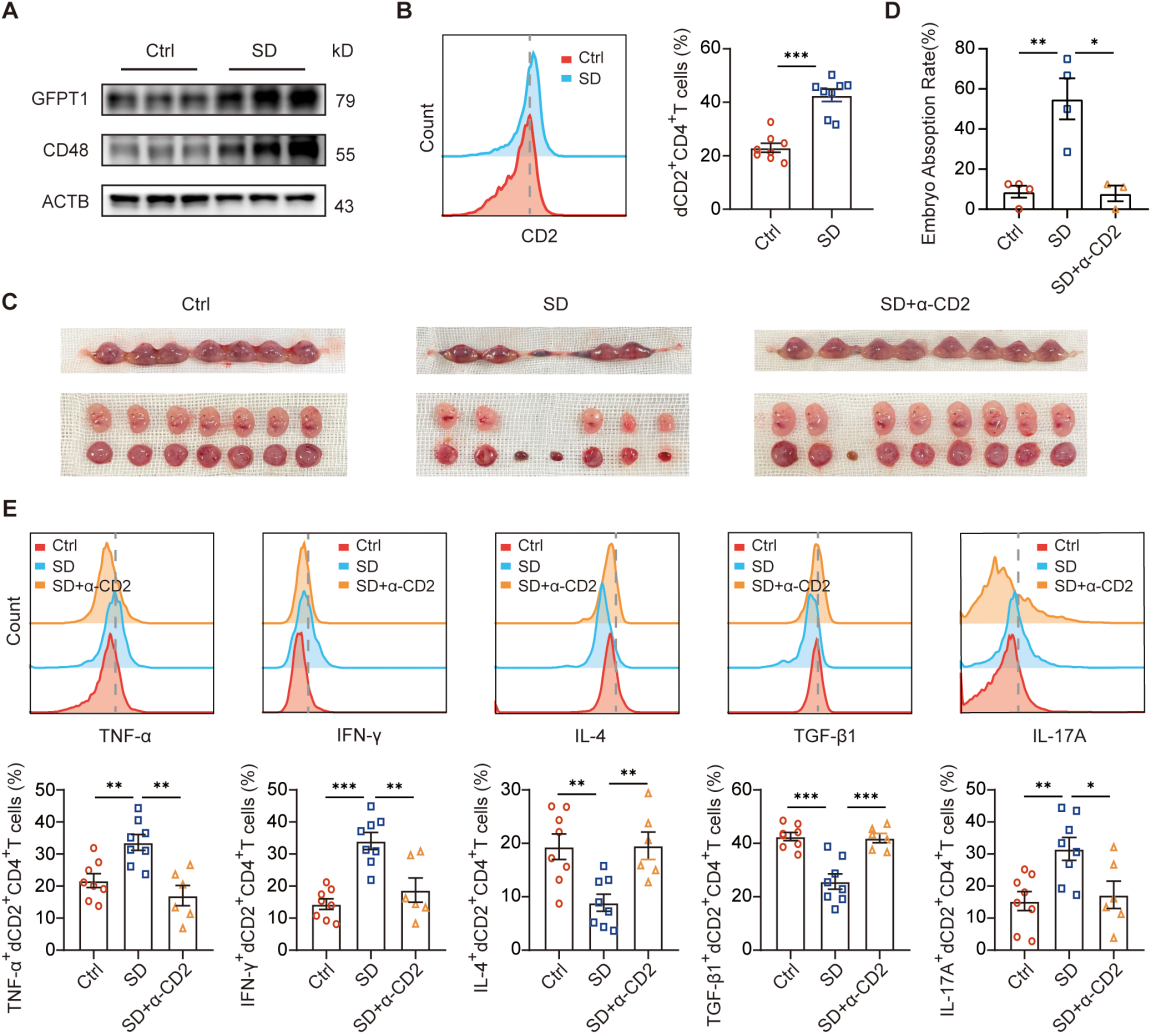
Effects of blockade of CD2 on pregnancy of mouse with SD. **(A)** The protein level of GFPT1 and CD48 on decidual tissue of pregnant mice with normal sleep (Ctrl) or sleep disturbance (SD). **(B)** Frequency of CD2 expressing cells in gated CD4^+^ T cells from decidual immune cells from Ctrl and SD mice as determined by flow cytometric analysis (n=8, Treatment groups were compared via Mann-Whitney-test). **(C, D)** The representative images of uterus **(C)** and the percentage of fetal resorption **(D)** of ctrl mice and SD mice treated with or without anti-CD2 antibody (Ctrl and SD group n=4, SD+α-CD2 group n=3, Treatment groups were compared via Student’s t-test). **(E)** Quantification of flow cytometric analysis of cytokine expression by dCD2^+^CD4^+^ T cells from Ctrl group and SD group treated with or without anti-CD2 antibody. Images are representative of three individual experiments (Ctrl and SD group n=8, SD+αCD2 group n=6, Treatment groups were compared via Student’s t-test). Data represent the mean ± SEM. The data points represent the biological replicates obtained from three independent experiments. *P<0.05, **P<0.01, ***p<0.001.

Next, we invested the role of Rev-erbα-GFPT1-CD58-CD2 axis on pregnancy outcome *in vivo*. We reported decreased Rev-erbα expression in DSCs from SD group (14). CD48 was considered the mouse counterpart of human CD58 (28). Here, higher GFPT1 and CD48 expression in decidual tissues were also observed in SD mice compared with that from normal pregnant mice (**Figure 5A**). We also observed more dCD2^+^CD4^+^T cells (**Figure 5B**), increased abortion rate (**Figures 5C, D**), and higher expression of Th1-type cytokines, but lower Th2- and Treg-type cytokines by dCD2^+^CD4^+^T cells (**Figure 5E**) in SD group. Treatment with CD2 blocking antibody significantly decreased the embryo resorption rate of SD mice (**Figures 5B, C**). Analysis of the dCD4^+^T cells from the treated mice revealed that TNF-α, IFN-γ and IL-17A expression of dCD2^+^CD4^+^T cells (**Figure 5E**) were also decreased, while IL-4, TGF-β1 expression of dCD2^+^CD4^+^T cells (**Figure 5E**) were increased. Taken together with our *in vitro* data, Rev-erbα-GFPT1-CD58-CD2 axis affected by sleep regulated the crosstalk between DSCs and dCD4^+^T cells so to play important role in the maintenance of pregnancy.

**Figure 6:**
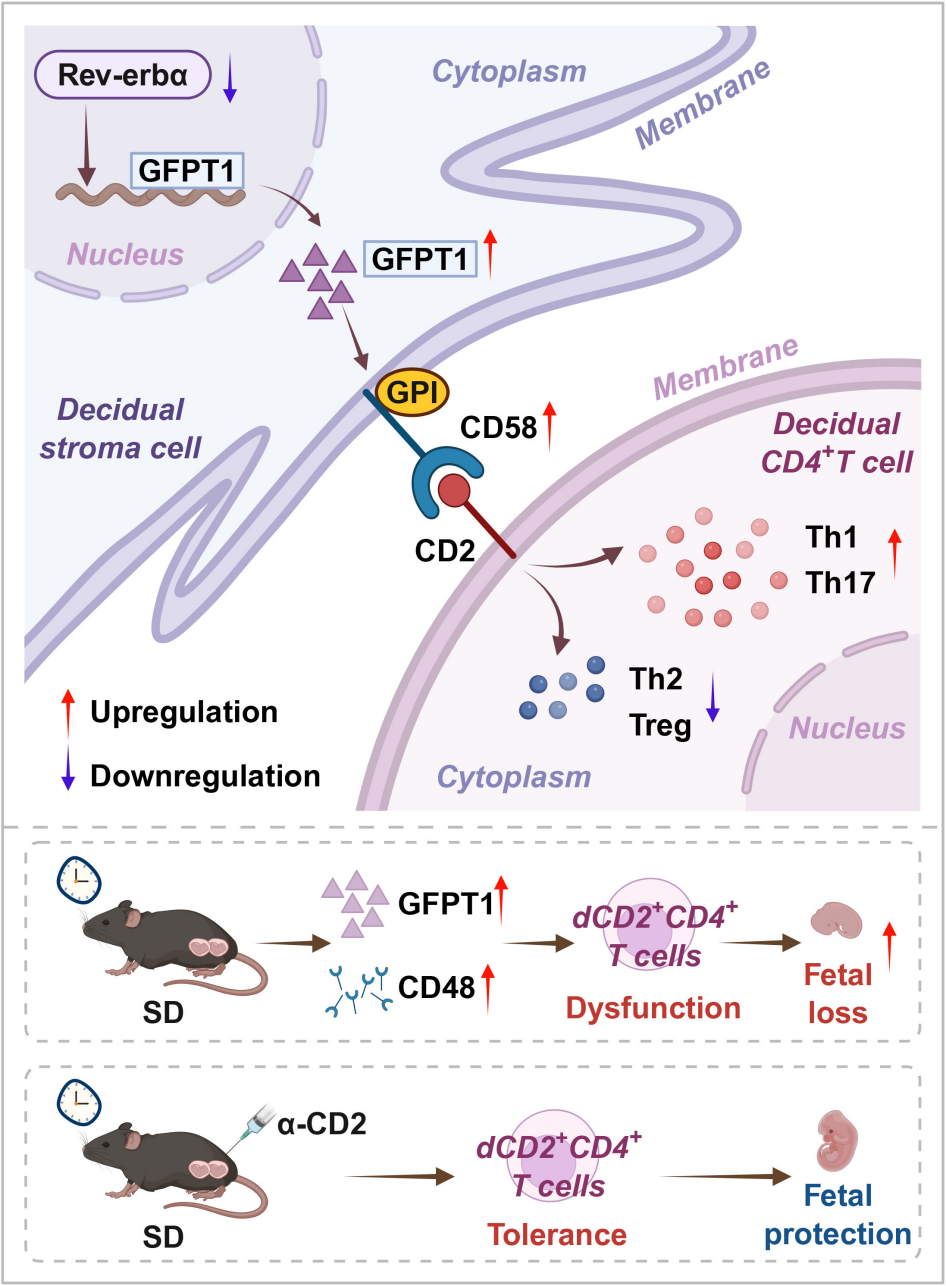
Schematic diagram of dysfunctional dCD2^+^CD4^+^T cells regulated by Rev-erbα-GFPT1-CD58 axis of DSCs in RS. Our integrated bioinformatics, proteomic, functional and model studies elucidated that GFPT1, which regulated by the circadian gene Rev-erbα, increased CD58 expression via GPI modification, leading to the disorder of dCD2^+^CD4^+^T cells and consequently, resulting in miscarriage. In addition, anti-CD2 antibody clearly decreased the risks of abortion of SD. This schematic clarified important implications for understanding the etiology of RPL and developing targeted therapy of RPL, especially those with sleep problems. RS, patient of RPL with sleep disturbance; SD, mice with sleep disturbance.

## 4 Discussion

Recurrent pregnancy loss (RPL) is a multifaceted condition wherein many clinical cases remain enigmatic. Women undergo significant changes in systems during pregnancy, which can profoundly affect their sleep patterns, and thus, poor sleep is a common problem during pregnancy (29). However, there are incomplete data on their implications for pregnancy outcomes though the literature on the effects of poor sleep on health in adults has received increasing scrutiny in recent years. Studies are still required on whether sleep disorders directly lead to RPL, especially unexplained RPL. Insomnia has been reported to contribute to spontaneous abortion (12). Epidemiological investigations showed that sleep disruption including chronic jet lag and work rotating shifts, fixed night shifts, or longer hours have an increased risk of adverse pregnancy outcomes such as miscarriage, infertility and preeclampsia (30–32). In addition, it would appear that first trimester sleep is actually quite important to birth outcomes, although many mothers report the most significant trouble sleeping in the third trimester (33). Sleep modulated the function of circadian genes and hormones, such as Per1, Bmal1, estradiol and melatonin, whose disruption are important risk factors for adverse pregnancy outcomes (31). Apoptosis in mouse embryonic stem cells could be induced via downregulation of circadian locomotor output cycles kaput (13). Dysfunction of the circadian rhythm period 1 could not synchronize aperiodic decidual gene expression with initiation of endometrial proliferation (34). Deficient decidualization, declined implantation number, and increased abortion rate were observed in the sleep disorder models (14). Thus sleep-related circadian disruption may serve as a modifiable risk factor for intervention throughout the preconception and pregnancy periods. In the future, accurate assessments of sleep such as validated self-reported measures via pittsburgh sleep quality index (PSQI) questionnaire or objective sleep measures via actigraphy (a wearable device designed to detect sleep and wake) may be necessary for women with poor sleep quality or history of RPL. The first recommended approach for women with sleep disorders is regular sleep scheduling and minimizing blue light exposure in the evening via avoiding screens one hour before bedtime. If the primary intervention is ineffective, referral to a sleep medicine center or cognitive behavioral therapy for insomnia is recommended. These management strategies may be helpful for sleep better and conducive to the maintenance of pregnancy for women with sleep disruption.

In the present study, the differentially expressed genes (DEGs) of endometrial stromal cells (ESCs) and decidualized ESCs (dESCs) were enriched in circadian rhythm and glycometabolism related signaling pathways. Previously, we reported lower Rev-erbα expression on decidual stromal cells (DSCs) from patients of RPL with sleep disturbance (RS) (14). Here, our omics analyses and functional experiments showed that the circadian gene Rev-erbα could regulate glycometabolism-related glutamine-fructose-6-phosphate amidotransferase 1 (GFPT1) expression of DSCs. Mutations in *GFPT1* were associated with congenital myasthenic syndromes and clinical worsening during pregnancy was common (35). High GFPT1 expression was also identified as a tumor promotor and an independent predictor of adverse clinical outcome for pancreatic ductal adenocarcinoma patients (36, 37). The inhibition of GFPT1 in cancer cells enhanced the activation of T cells and the cancer-killing activity of NK cells (38). The differentially expressed proteins between DSCs with and without *GFPT1* knockdown were enriched in immune regulation related pathways, suggesting that GFPT1 signaling might also take part in regulating maternal-fetal tolerance. We further confirmed that GFPT1 increased CD58 expression of DSCs. This CD58 upregulation cascaded into dCD4^+^T cell dysfunction via CD2, and might ultimately, fetal loss in RS.

As hexosamine biosynthesis pathway rate-limiting enzyme, GFPT1 was profoundly implicated in the classical O-linked and N-linked glycosylation (25, 26). Protein glycosylation as a common post-translational modification that has significant impacts on protein expression and function. However, inhibition of N-linked glycosylation and O-linked glycosylation had no effect on CD58 expression of DSCs. We found that GFPT1 increased CD58 expression via GPI modification. GPI anchoring is a conserved post-translational modification which allows proteins to be expressed at the cell surface as GPI anchored proteins (39). *GFPT1* mutant led to the rapid depletion of GPI precursors (27). Although we detected glycosylphosphatidylinositol (GPI) anchored CD58 by phosphatidylinositol-specific phospholipase C (PI-PLC) sensitivity assay, PI-PLC sensitivity indicates GPI anchoring but does not distinguish between increased surface stability versus altered trafficking or degradation. Cycloheximide chase assay showed that GFPT1 knockdown in DSCs promoted CD58 protein degradation. However, the regulatory mechanism of GFPT1 on GPI-CD58 stabilization or trafficking remained unclear. Uridine diphosphate (UDP)-N-acetylglucosamine (GlcNAc) was an important product of hexosamine pathway catalyzed by GFPT1. In GPI precursor synthesis, GlcNAc from UDP - N - GlcNAc was transferred to the 6-position of inositol to generate GlcNAc-PI and then assembled to GPI anchored proteins (GPI-APs) (40). If the precursor proteins of GPI-APs are not processed by GPI transamidase for GPI attachment, they will be degraded (41). GPI-APs was translocated to plasmic membrane after going through several remodelling steps in the endoplasmic reticulum and the golgi apparatus, which were regulated by many enzymes. Thus, GFPT1 may regulate GPI-CD58 stabilization by regulating GPI synthesis or the activity of these enzymes in the process of mature and translocation of GPI-APs. Further research is needed to determine the specific mechanisms in the future.

CD4^+^T cells are thought to play a pivotal role in maternal-fetal tolerance and for controlling maternal viremia (42). The production of pro- or anti-inflammatory cytokines at the maternal-fetal interface influences the outcome of pregnancy because there is dominance by Th2- and Treg-type cytokines during normal pregnancy and dominance by Th1- and Th17-type cytokines in RPL (43). CD2 is mainly expressed on the surface of T cells, especially on effective T cells, which is essential molecule for T cell activation (44). We found that dCD4^+^T cells were characterized by differential expression of CD2 as dCD2^+^CD4^+^T cells expressed more Th1- and Th17-type cytokines than dCD2^-^CD4^+^T cells. CD58-CD2 interactions on T cells enabled more robust T cell receptor (TCR) signals to deliver co-stimulation signal during T cell activation (28, 45), whose excessive activation disrupts immune tolerance in pregnancy. However, immune checkpoints such as cytotoxic T-lymphocyte antigen 4 (CTLA-4), T-cell immunoglobulin and mucin-domain containing- 3 (Tim-3), programmed death receptor 1 (PD-1) and lymphocyte activation gene 3 (LAG-3) are highly expressed at maternal-fetal interface, whose activation promote decidual Th2 or Treg cytokine production and are beneficial for maternal-fetal immune tolerance (46). J E Woodward et al. suggested that anti-CD2 plus CTLA-4 Ig are more efficient in inducing immune tolerance than monotherapy (47). Defects in CD58-CD2 interaction diminished T cell activation and increased PD-L1 protein stabilization (48). Whether CD2 regulated function of CD4^+^ T cell by inhibiting expression of immune checkpoints at maternal-fetal interface remains to be proved in the future. These will inspire us to consider whether simultaneously regulating Rev-erbα-GFPT1-CD58-CD2 axis integrating with immune checkpoints are more effective for the treatment of miscarriage.

Previous research suggested that anti-CD2 preferentially targets CD2^hi^ T cells (49) and differences in CD2 expression might determine the response to anti-CD2 antibody (45). CD2 is strongly upregulated in the dCD4^+^T cells of RS patients and mice with sleep disturbance (SD) models. CD2 blockade resulted in the decreased production of pro-inflammatory cytokines of dCD2^+^CD4^+^T cells both *in vivo* and *in vitro*. Furthermore, CD2 blocking antibody alleviated the adverse effect of sleep disruption on pregnancy outcomes. These data indicated that the upregulation in proportion and the abnormity in functionality of dCD2^+^CD4^+^T cells, which were regulated by Rev-erbα-GFPT1-CD58 axis of DSCs, were associated with imbalance of maternal-fetal immunity and fetal loss of SD mice. CD2 has been explored as a therapeutic target in several autoimmune diseases (50, 51). Some research indicated that anti-CD2 might induce exhaustion of memory CD4^+^ T subsets (52). The duration and concentration of medication may affect the degree of exhaustion. Although the present study suggested a promising prospect for CD2 as a therapeutic target for RS patients, we need to pay attention to the side effects of systemic immune suppression of anti-CD2 in the future.

## 5 Conclusions

In summary, the present study showed that GFPT1, which regulated by the circadian gene Rev-erbα, increased CD58 expression via GPI modification, leading to the disorder of dCD2^+^CD4^+^T cells and consequently, resulting in miscarriage of SD. In addition, CD2 blocking antibody clearly decreased the risks of abortion of SD mice, proving a potential therapeutic target for adverse pregnancy outcomes induced by circadian rhythm disruption. Our findings highlighted the association between poor sleep and adverse pregnancy outcomes, further indicating that we should pay attention to the sleep health of pregnant women, especially those with a history of RPL.

## Data Availability

The original contributions presented in the study are included in the article/**Supplementary Material**. Further inquiries can be directed to the corresponding authors.

## Supplementary material

Supplementary Figure 1 Cycloheximide (CHX) chase assay showed the increased degradation of CD58 in DSCs with GFPT1 knockdown. Images are representative of three individual experiments.

## References

[1] Dimitriadis E Menkhorst E Saito S Kutteh WH Brosens JJ . Recurrent pregnancy loss. Nat Rev Dis Primers. (2020) 6:98. doi: 10.1038/s41572-020-00228-z, PMID: 33303732

[2] Shennan AH Story L . Cervical cerclage: green-top guideline no. 75. BJOG: An Int J Obstetr Gynaecol. (2022) 129:1178–210. doi: 10.1111/1471-0528.17003, PMID: 35199905

[3] Rasmark Roepke E Christiansen OB Källén K Hansson SR . Women with a history of recurrent pregnancy loss are a high-risk population for adverse obstetrical outcome: A retrospective cohort study. J Clin Med. (2021) 10:179. doi: 10.3390/jcm10020179, PMID: 33419111 PMC7825424

[4] Hautamäki H Gissler M Heikkinen-Eloranta J Tiitinen A Peuranpää P . Pregnancy and perinatal outcomes in women with recurrent pregnancy loss-A case-control study. Acta Obstetr Gynecol Scandinavica. (2025) 104:368–79. doi: 10.1111/aogs.15039, PMID: 39711128 PMC11782061

[5] Bagkou Dimakou D Lissauer D Tamblyn J Coomarasamy A Richter A . Understanding human immunity in idiopathic recurrent pregnancy loss. Eur J Obstetr Gynecol Reprod Biol. (2022) 270:17–29. doi: 10.1016/j.ejogrb.2021.12.024, PMID: 35007974

[6] Cha J Sun X Dey SK . Mechanisms of implantation: strategies for successful pregnancy. Nat Med. (2012) 18:1754–67. doi: 10.1038/nm.3012, PMID: 23223073 PMC6322836

[7] Kojima J Ono M Kuji N Nishi H . Human chorionic villous differentiation and placental development. Int J Mol Sci. (2022) 23:8003. doi: 10.3390/ijms23148003, PMID: 35887349 PMC9325306

[8] Arck PC Hecher K . Fetomaternal immune cross-talk and its consequences for maternal and offspring’s health. Nat Med. (2013) 19:548–56. doi: 10.1038/nm.3160, PMID: 23652115

[9] Haram K Mortensen JH Myking O Roald B Magann EF Morrison JC . Early development of the human placenta and pregnancy complications. J Matern Fetal Neonatal Med. (2020) 33:3538–45. doi: 10.1080/14767058.2019.1578745, PMID: 30810433

[10] Ng SW Norwitz GA Pavlicev M Tilburgs T Simón C Norwitz ER . Endometrial decidualization: the primary driver of pregnancy health. Int J Mol Sci. (2020) 21:4092. doi: 10.3390/ijms21114092, PMID: 32521725 PMC7312091

[11] Garmendia JV De Sanctis CV Hajdúch M De Sanctis JB . Exploring the immunological aspects and treatments of recurrent pregnancy loss and recurrent implantation failure. Int J Mol Sci. (2025) 26:1295. doi: 10.3390/ijms26031295, PMID: 39941063 PMC11818386

[12] He Y Wang L Tang R Jin H Liu B Chen S . Common mental disorders and risk of spontaneous abortion or recurrent spontaneous abortion: A two-sample Mendelian randomization study. J Affect Disord. (2024) 354:258–66. doi: 10.1016/j.jad.2024.03.026, PMID: 38484879

[13] Li R Cheng S Wang Z . Circadian clock gene plays a key role on ovarian cycle and spontaneous abortion. Cell Physiol Biochem: Int J Exp Cell Physiol Biochem Pharmacol. (2015) 37:911–20. doi: 10.1159/000430218, PMID: 26390085

[14] Cui L Xu F Xu C Ding Y Wang S Du M . Circadian gene Rev-erbα influenced by sleep conduces to pregnancy by promoting endometrial decidualization via IL-6-PR-C/EBPβ axis. J BioMed Sci. (2022) 29:101. doi: 10.1186/s12929-022-00884-1, PMID: 36419076 PMC9685872

[15] Cui L Xu F Wang S Li X Lin H Ding Y . Pharmacological activation of rev-erbα suppresses LPS-induced macrophage M1 polarization and prevents pregnancy loss. BMC Immunol. (2021) 22:57. doi: 10.1186/s12865-021-00438-4, PMID: 34399700 PMC8369701

[16] Huang RF Chen JH Zhou MY Xin HR Lam SM Jiang XQ . Multi-omics profiling reveals rhythmic liver function shaped by meal timing. Nat Commun. (2023) 14:6086. doi: 10.1038/s41467-023-41759-9, PMID: 37773240 PMC10541894

[17] Yang Y Zhang DD Qin HM Liu S Yan Q . poFUT1 promotes endometrial decidualization by enhancing the O-fucosylation of Notch1. Ebiomedicine. (2019) 44:563–73. doi: 10.1016/j.ebiom.2019.05.027, PMID: 31201143 PMC6606927

[18] Chen S Zhang A Li N Wu H Li Y Liu S . Elevated high-mannose N-glycans hamper endometrial decidualization. iScience. (2023) 26:108170. doi: 10.1016/j.isci.2023.108170, PMID: 37915610 PMC10616321

[19] Zhong J Li J Burton GJ Koistinen H Cheung KW Ng EHY . The functional roles of protein glycosylation in human maternal-fetal crosstalk. Hum Reprod Update. (2024) 30:81–108. doi: 10.1093/humupd/dmad024, PMID: 37699855

[20] Zhou WJ Yang HL Mei J Chang KK Lu H Lai ZZ . Fructose-1,6-bisphosphate prevents pregnancy loss by inducing decidual COX-2(+) macrophage differentiation. Sci Adv. (2022) 8:eabj2488. doi: 10.1126/sciadv.abj2488, PMID: 35196096 PMC8865779

[21] Cui L Sun F Xu Y Li M Chen L Chen C . Tim-3 coordinates macrophage-trophoblast crosstalk via angiogenic growth factors to promote pregnancy maintenance. Int J Mol Sci. (2023) 24:1538. doi: 10.3390/ijms24021538, PMID: 36675047 PMC9867110

[22] Adlanmerini M Lazar MA . The REV-ERB nuclear receptors: timekeepers for the core clock period and metabolism. Endocrinology. (2023) 164:bqad069. doi: 10.1210/endocr/bqad069, PMID: 37149727 PMC10413432

[23] Wei S Zhao Q Zheng K Liu P Sha N Li Y . GFAT1-linked TAB1 glutamylation sustains p38 MAPK activation and promotes lung cancer cell survival under glucose starvation. Cell Discov. (2022) 8:77. doi: 10.1038/s41421-022-00423-0, PMID: 35945223 PMC9363421

[24] Zhang Y Liu Q Yang S Liao Q . CD58 immunobiology at a glance. Front Immunol. (2021) 12:705260. doi: 10.3389/fimmu.2021.705260, PMID: 34168659 PMC8218816

[25] Gélinas R Mailleux F Dontaine J Bultot L Demeulder B Ginion A . AMPK activation counteracts cardiac hypertrophy by reducing O-GlcNAcylation. Nat Commun. (2018) 9:374. doi: 10.1038/s41467-017-02795-4, PMID: 29371602 PMC5785516

[26] Paneque A Fortus H Zheng J Werlen G Jacinto E . The hexosamine biosynthesis pathway: regulation and function. Genes (Basel). (2023) 14:933. doi: 10.3390/genes14040933, PMID: 37107691 PMC10138107

[27] Naderer T Wee E McConville MJ . Role of hexosamine biosynthesis in Leishmania growth and virulence. Mol Microbiol. (2008) 69:858–69. doi: 10.1111/j.1365-2958.2008.06314.x, PMID: 18532982

[28] Jo Y Sim HI Yun B Park Y Jin HS . Revisiting T-cell adhesion molecules as potential targets for cancer immunotherapy: CD226 and CD2. Exp Mol Med. (2024) 56:2113–26. doi: 10.1038/s12276-024-01317-9, PMID: 39349829 PMC11541569

[29] Chang Y Sun Z Ning F Dang X Zhang G Tang J . Association between sleep disturbances during pregnancy and adverse perinatal outcomes. Am J Trans Res. (2024) 16:3886–96. doi: 10.62347/yxbm9408, PMID: 39262762 PMC11384389

[30] Zhu JL Hjollund NH Olsen J . Shift work, duration of pregnancy, and birth weight: the National Birth Cohort in Denmark. Am J Obstet Gynecol. (2004) 191:285–91. doi: 10.1016/j.ajog.2003.12.002, PMID: 15295380

[31] Kloss JD Perlis ML Zamzow JA Culnan EJ Gracia CR . Sleep, sleep disturbance, and fertility in women. Sleep Med Rev. (2015) 22:78–87. doi: 10.1016/j.smrv.2014.10.005, PMID: 25458772 PMC4402098

[32] Willis SK Hatch EE Wise LA . Sleep and female reproduction. Curr Opin Obstet Gyn. (2019) 31:222–7. doi: 10.1097/Gco.0000000000000554, PMID: 31082843

[33] Sweet L Arjyal S Kuller JA Dotters-Katz S . A review of sleep architecture and sleep changes during pregnancy. Obstet Gynecol Surv. (2020) 75:253–62. doi: 10.1097/ogx.0000000000000770, PMID: 32324251

[34] Muter J Lucas ES Chan YW Brighton PJ Moore JD Lacey L . The clock protein period 2 synchronizes mitotic expansion and decidual transformation of human endometrial stromal cells. FASEB J. (2015) 29:1603–14. doi: 10.1096/fj.14-267195, PMID: 25573754 PMC4396614

[35] O’Connell K Rooney T Alabaf S Ramdas S Beeson D Palace J . Pregnancy outcomes in patients with congenital myasthenic syndromes. Muscle Nerve. (2022) 66:345–8. doi: 10.1002/mus.27653, PMID: 35661384

[36] Jia C Li H Fu D Lan Y . GFAT1/HBP/O-glcNAcylation axis regulates β-catenin activity to promote pancreatic cancer aggressiveness. BioMed Res Int. (2020) 2020:1921609. doi: 10.1155/2020/1921609, PMID: 32149084 PMC7048922

[37] Yang C Peng P Li L Shao M Zhao J Wang L . High expression of GFAT1 predicts poor prognosis in patients with pancreatic cancer. Sci Rep. (2016) 6:39044. doi: 10.1038/srep39044, PMID: 27996048 PMC5172351

[38] Chen W Saxton B Tessema M Belinsky SA . Inhibition of GFAT1 in lung cancer cells destabilizes PD-L1 protein. Carcinogenesis. (2021) 42:1171–8. doi: 10.1093/carcin/bgab063, PMID: 34270713 PMC8491135

[39] Hirayama H . Commentary for: a lipid scramblase TMEM41B is involved in the processing and transport of GPI-anchored proteins. J Biochem. (2025) 177:69–71. doi: 10.1093/jb/mvae085, PMID: 39658195

[40] Kinoshita T . Biosynthesis and biology of mammalian GPI-anchored proteins. Open Biol. (2020) 10:190290. doi: 10.1098/rsob.190290, PMID: 32156170 PMC7125958

[41] Ashok A Hegde RS . Retrotranslocation of prion proteins from the endoplasmic reticulum by preventing GPI signal transamidation. Mol Biol Cell. (2008) 19:3463–76. doi: 10.1091/mbc.E08-01-0087, PMID: 18508914 PMC2488287

[42] Bialas KM Tanaka T Tran D Varner V Cisneros de la Rosa E Chiuppesi F . Maternal CD4+ T cells protect against severe congenital cytomegalovirus disease in a novel nonhuman primate model of placental cytomegalovirus transmission. Proc Natl Acad Sci U S A. (2015) 112:13645–50. doi: 10.1073/pnas.1511526112, PMID: 26483473 PMC4640765

[43] Wang S Chen C Li M Qian J Sun F Li Y . Blockade of CTLA-4 and Tim-3 pathways induces fetal loss with altered cytokine profiles by decidual CD4(+)T cells. Cell Death Dis. (2019) 10:15. doi: 10.1038/s41419-018-1251-0, PMID: 30622243 PMC6325160

[44] Lebrec H Bui J Clingan JM Do J Dubovsky J Dragone L . Second generation CD2-targeting LFA-3 fusion protein SBT115301 to restore immune homeostasis in autoimmune disease. Iscience. (2025) 28:112447. doi: 10.1016/j.isci.2025.112447, PMID: 40491961 PMC12146614

[45] Fernandez Lahore G Förster M Johannesson M Sabatier P Lönnblom E Aoun M . Polymorphic estrogen receptor binding site causes Cd2-dependent sex bias in the susceptibility to autoimmune diseases. Nat Commun. (2021) 12:5565. doi: 10.1038/s41467-021-25828-5, PMID: 34552089 PMC8458462

[46] Hu XH Lai SY Liao AH . Immune checkpoint for pregnancy. Semin Immunopathol. (2025) 47:26. doi: 10.1007/s00281-025-01051-y, PMID: 40314833

[47] Woodward JE Qin LH Chavin KD Lin JX Tono T Ding YZ . Blockade of multiple costimulatory receptors induces hyporesponsiveness - Inhibition of CD2 plus CD28 pathways. Transplantation. (1996) 62:1011–8. doi: 10.1097/00007890-199610150-00021, PMID: 8878397

[48] Ho PTC Melms JC Rogava M Frangieh CJ Pozniak J Shah SB . The CD58-CD2 axis is co-regulated with PD-L1 via CMTM6 and shapes anti-tumor immunity. Cancer Cell. (2023) 41:1207. doi: 10.1016/j.ccell.2023.05.014, PMID: 37327789 PMC10524902

[49] Lo DJ Weaver TA Stempora L Mehta AK Ford ML Larsen CP . Selective targeting of human alloresponsive CD8+ effector memory T cells based on CD2 expression. Am J Transpl: Off J Am Soc Transplant Am Soc Transplant Surgeons. (2011) 11:22–33. doi: 10.1111/j.1600-6143.2010.03317.x, PMID: 21070604 PMC3057516

[50] Binder C Sellberg F Cvetkovski F Berglund E Berglund D . Siplizumab, an anti-CD2 monoclonal antibody, induces a unique set of immune modulatory effects compared to alemtuzumab and rabbit anti-thymocyte globulin. In Vitro Front Immunol. (2020) 11:592553. doi: 10.3389/fimmu.2020.592553, PMID: 33262770 PMC7686512

[51] Rostaing L Charpentier B Glyda M Rigotti P Hettich F Franks B . Alefacept combined with tacrolimus, mycophenolate mofetil and steroids in *de novo* kidney transplantation: a randomized controlled trial. Am J Transpl: Off J Am Soc Transplant Am Soc Transplant Surgeons. (2013) 13:1724–33. doi: 10.1111/ajt.12303, PMID: 23730730

[52] Berglund E Alonso-Guallart P Danton M Sellberg F Binder C Fröbom R . Safety and pharmacodynamics of anti-CD2 monoclonal antibody treatment in cynomolgus macaques - an experimental study. Transpl Int. (2020) 33:98–107. doi: 10.1111/tri.13524, PMID: 31523849 PMC7017722

