# Supplementary figures and images for: Dysfunctional decidual CD2+CD4+T cells regulated by Rev-erbα - GFPT1 - GPI anchored CD58 axis of decidual stromal cells underlies sleep disturbance induced recurrent pregnancy loss
Source: Front Immunol. 2026 Feb 18;17:1703925. doi: 10.3389/fimmu.2026.1703925 (PMC12956714; doi:10.3389/fimmu.2026.1703925)

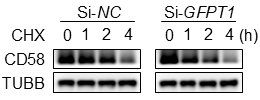

Supplement: Supplementary Figure 1 — Cycloheximide (CHX) chase assay showed the increased degradation of CD58 in DSCs with GFPT1 knockdown. Images are representative of three individual experiments. [file Image1.tif]
